# A mechanistic study of gold nanoparticle radiosensitisation using targeted microbeam irradiation

**DOI:** 10.1038/srep44752

**Published:** 2017-03-16

**Authors:** Mihaela Ghita, Stephen J. McMahon, Laura E. Taggart, Karl T. Butterworth, Giuseppe Schettino, Kevin M. Prise

**Affiliations:** 1 Centre for Cancer Research and Cell Biology, Queen’s University Belfast, 97 Lisburn Road, BT7 9AE, Belfast, UK; 2 Department of Radiation Oncology, Massachusetts General Hospital and Harvard Medical School, Boston, MA 02114, USA; 3 National Physics Laboratory, Teddington, TW11 0LW, London, UK

## Abstract

Gold nanoparticles (GNPs) have been demonstrated as effective radiosensitizing agents in a range of preclinical models using broad field sources of various energies. This study aimed to distinguish between these mechanisms by applying subcellular targeting using a soft X-ray microbeam in combination with GNPs. DNA damage and repair kinetics were determined following nuclear and cytoplasmic irradiation using a soft X-ray (carbon K-shell, 278 eV) microbeam in MDA-MB-231 breast cancer and AG01522 fibroblast cells with and without GNPs. To investigate the mechanism of the GNP induced radiosensitization, GNP-induced mitochondrial depolarisation was quantified by TMRE staining, and levels of DNA damage were compared in cells with depolarised and functional mitochondria. Differential effects were observed following radiation exposure between the two cell lines. These findings were validated 24 hours after removal of GNPs by flow cytometry analysis of mitochondrial depolarisation. This study provides further evidence that GNP radiosensitisation is mediated by mitochondrial function and it is the first report applying a soft X-ray microbeam to study the radiobiological effects of GNPs to enable the separation of physical and biological effects.

Radiotherapy has rapidly progressed in recent decades and has become one of the most therapeutically and cost effective tools in cancer treatment. While the key driver of radiotherapy developments is the delivery of improved physical dose distributions^1^, there is increasing interest in the combination of sophisticated dose delivery techniques with new imaging tools introducing new concepts as ‘biological target volume’, ‘molecular imaging’ and ‘theranostics’ to radiobiologically targeted therapy^2^.

Interest in the use of radiotherapy contrast agents was stimulated by early studies finding elevated levels of damage in tissues after contrast enhanced medical imaging, indicating that the presence of a high-Z material can increase radiation damage^3^. This is attributed to the high photoelectric cross-section of these materials which means that high Z materials absorb substantially more energy per unit mass than soft tissue (between 10–150 times for kV photons) and which translates to an increase in local dose^4^. While these dose enhancing effects were undesirable in imaging, there was interest in applying them to improve tumour cell killing in therapy.

Early attempts were made to achieve radiation dose enhancements using gold (Z = 79) in the form of foil or microspheres^5^ as pioneering work for the use of gold as a radiosensitisers, but this was limited by delivery challenges. However, nanoparticles (NPs) have been shown^6^ to inherently become trapped in tumour tissues due to the poorly formed leaky tumour vasculature allowing nanoparticles to pass into the tumour volume and become internalised. This specific feature gives Gold Nanoparticles (GNPs) the potential to be used as tumour-specific radiosensitisers either directly^6^ or by modifying them with tumour-targeting antibodies^7^.

The effectiveness of these particles was initially explained purely in terms of dose-modifying effects. Previous work^8^ has calculated the amount of GNPs required within a tumour to significantly enhance dose deposition based on the ratio of the mass energy absorption coefficients of gold and soft tissue. Calculations have suggested delivering 1% of gold by mass to the tumour would mean the doubling of the amount of energy deposited locally by a 160–225 kV X-ray source. There are an increasing number of studies revolving around the interactions between radiation gold and soft tissue for a variety of configurations of source and target geometry, suggesting that GNPs offer enhanced control over radiation effects and dose boosts localised within the tumour volume^8^,^9^.

However, experimental investigations of GNP radiosensitization have shown very poor correlation with these physical dose predictions, in many cases seeing significantly greater sensitisation than predicted^4^. It has been suggested that these effects are driven either through more complex sub-cellular changes to dose distributions, or through biological effects.

When GNPs interact with energetic ionising radiation, a cascade of low energy, short range secondary particles are produced, depositing very high doses within the immediate vicinity of the nanoparticle^10^, which have been suggested to drive greater biological effects. Since cell death from ionising radiation is generally initiated by damage to the DNA molecule, the short-range effect of GNPs emphasises the importance of sub-cellular uptake and localisation. It is often assumed that GNPs need to be placed within the cell nucleus to destroy cancer cells, prompting significant efforts to develop coatings to facilitate GNP nuclear internalisation.

However, previous experimental studies have shown that GNPs tend to be taken up in the cytoplasm of the cell^11^ and yet may still have a dose enhancing effect^12^. Whether this can be attributed in part to a biological mechanism or to physical processes such as radiation damage to extra-nuclear targets is still not known^13^. In a recent study of 1.9 nm GNPs, strong radiosensitization has been observed in long term cell survival with no nuclear localisation of GNPs^13^. The same work demonstrated DNA damage present in the cells as a consequence of GNP treatment only, with subsequent irradiation resulting in no additional difference between treated and untreated cells. This work suggested that mitochondria may play an important role in driving biological responses to these GNPs. However, separating these effects from radiation-induced damage remains challenging, due to the potential of ‘crosstalk’ where radiation scatters from particles within the cytoplasm and damages nuclear targets.

The efficacy of Aurovist 1.9 nm Au nanoparticles has been established in previous work through clonogenic cell survival assays in various cancer cell lines^14^. While previous work has focused on elucidating the chemical^13^ and modelling the physical^15^ interactions of these GNPs with cells, the mechanism and differences between the cancer and normal cell lines is still under debate with a mitochondrial mediated mechanism being proposed^13^,^16^,^17^. This work aims to isolate the biological effect at the cellular level for Aurovist nanoparticles.

This is achieved by using very low energy irradiation targeted to specific cell compartments while monitoring the mitochondrial status. An ultrasoft X-ray microbeam is used for this work, allowing quantification of DNA damage and repair after irradiation targeted to either the cell cytoplasm or nucleus, with or without GNPs. The X-ray microprobe makes use of 278 eV carbon K-shell X-rays, enabling very precise target accuracy and dose delivery^18^,^19^,^20^. Importantly, this energy is much lower than that used in most radiobiology experiments, producing secondary electrons with a range of only a few nanometres. This ensures that no secondary particles produced by radiation interactions within the cytoplasm can cause damage within the nucleus, allowing a separation of physical and biological effects.

## Materials and Methods

### Cell culture

Human primary fibroblast AG01522 cells were obtained from the Coriell Institute for Medical Research (Camden, NJ, USA) and maintained in α-modified Eagle’s Medium (Lonza, UK) supplemented with 20% Fetal Bovine Serum (PAA Laboratories, UK) and 1% Penicillin/Streptomycin (Sigma, UK). Breast adenocarcinoma MDA-MB-231 cells obtained from Cancer Research UK were grown in Dulbeco’s Modified Essential Media (Sigma, UK) supplemented with 10% FBS and 1% Penicilin/Streptomycin. All cell lines were maintained at 37 °C in a humidified atmosphere of 5% CO_2_. One day prior to microbeam irradiation 10^5^ cells were seeded onto specially designed 0.5 μm Mylar^®^ base microbeam dishes with 2 ml of media and allowed to adhere overnight as previously described^18^.

Before irradiation the cell cytoplasm was stained with 0.1 μg/mL Nile Red (Sigma, UK) and incubated for 10 minutes; the nucleus was stained with 0.2 μg/mL Hoechst 33342 (Sigma, UK) for 30 minutes before irradiation. This enabled the cytoplasm and nucleus to be identified using fluorescent microscopy on the microbeam system.

### Gold nanoparticles

1.9 nm Aurovist™ are spherical gold particles with a proprietary thiol coating^14^ purchased from Nanoprobes Inc. (NY, USA) and re-suspended in sterile water. The treated cells were incubated prior to irradiation for 24 hours with 500 μg/mL GNPs. For this specific type of nanoparticles with a 1.9 nm diameter 500 μg/mL will mean a concentration of 12 μM. This concentration and incubation time for GNPs were chosen as a result of previous work within the group showing that these conditions allow for optimal cell uptake of GNPs^14^.

On the day of the experiment, GNP containing media was removed from the cells, being replaced with fresh supplemented media 15 minutes before irradiation. The concentration and incubation time were chosen as a result of previous work^13^ showing these conditions allow for optimal cell uptake of GNPs^14^.

### Microbeam irradiation

The soft X-ray source was used to generate a micrometer sized beam in order to irradiate specific parts of the cell. Mechanisms of carbon characteristic X-ray (278 eV) production, specifics of the microprobe source and standard cell irradiation procedure have been described elsewhere^18^,^21^. All cells were irradiated with 2 ± 0.2 Gy to the targeted region, the dose being calculated as the energy deposited by absorbed photons relative to the nucleus mass as reported by ICRU 46 (1992) report^22^. When calculating the deposited energy the detector efficiency of 99% for the photomultiplier chamber was considered for this specific energy and the unfocused photon contribution was considered to be 0.45% of the total dose^18^. Control non-irradiated dishes with and without GNPs were scanned with the microbeam system and then incubated and fixed.

**Figure 1:**
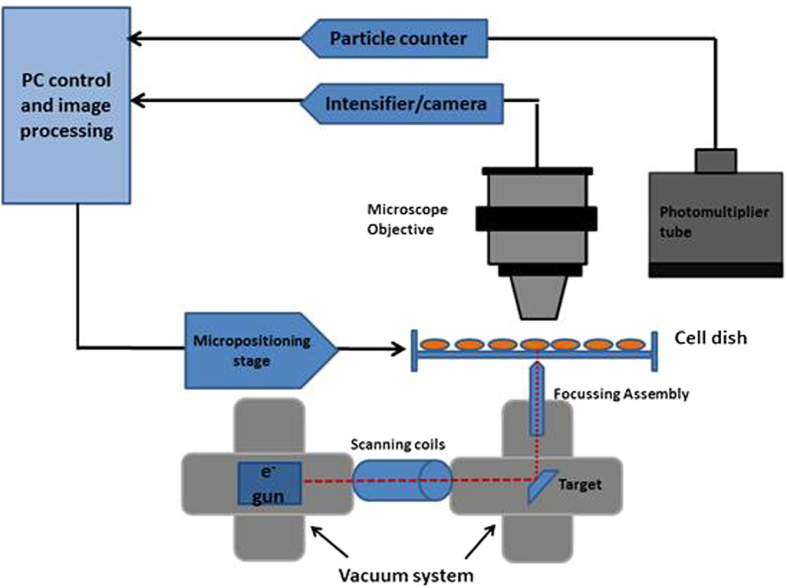
Schematic of the soft X-ray microbeam setup showing the beam path from the electron gun to the specimen dish and the microscope and particle counter setup above the stage.

A microbeam spot of 5 μm diameter with a dose rate of 0.15 ± 0.015 Gy/sec was used to deliver carbon K-shell X-ray to the nucleus or cytoplasm with and without GNPs. Before irradiation, cells were stained as described above and localized under microscopic view using low level UV illumination. Figure 1 shows a schematic representation of the irradiation setup for this study.

### Inductively coupled plasma mass spectrometry

1.5 × 10^5^ cells were plated for 6 hours then incubated with 1.9 nm GNPs for 24 hours. Upon completion of the time course they were washed in PBS, trypsinised, counted and digested in 0.5 ml aqua regia (1 part 100% nitric acid: 3 parts 100% hydrochloric acid). Each sample volume was made up to 5 ml with distilled water and gold content was determined using a Perkin Elmer Optima 4300 DV Inductively Coupled Plasma Optical Emission Spectrometer. The number of GNPs per cell was determined as previously described^23^.

### Fluorescent-lifetime imaging microscopy (FLIMS)

Cells were seeded onto sterile 16 mm^2^ round coverslips placed in six well plates at a density of 1 × 10^5^ cells per well. Cells were left to attach for 4–6 hours before treatment with 500 μg/mL GNPs for 24 hours. Cells were then fixed with a solution of 50% acetone and 50% methanol for ten minutes before being washed with PBS and nuclei stained with DAPI (20 μg/mL) for 10 minutes. The DAPI was removed and the cells washed with PBS twice before being mounted onto glass microscope slides with 5 μl of Vectashield mounting media (Vector Labs Ltd, UK). Cells were imaged by multiphoton fluorescence lifetime imaging Microscopy (MP-FLIM) using a confocal microscope part of OCTOPUS imaging cluster at the Research Complex in Harwell^24^. Conventional confocal microscopy was used to image DAPI stain with a 405 ± 20 nm excitation laser and a 450 ± 20 nm filter. The imaging procedure can be used to produce an image of the subcellular gold distribution since the fast component corresponds to the decay of the LSPR (Localised Surface Plasmon Resonance) of the GNPs, while the slow component arises from the non-linear excitation of the cytoplasm or nuclear DNA as previously described^25^.

### DNA damage assay

After irradiation dishes were incubated for 1, 3, 6 and 24 hours and then fixed with 4% paraformaldehyde (Sigma, UK). Non irradiated controls were incubated for 3–6 hours. Cells were then stained as previously described^26^.

DNA damage was measured by revisiting the irradiated cells using the coordinate recording function on the microbeam microscope stage and counting 53BP1 foci. Experiments were performed in at least 3 independent repeats, with at least 30 cells irradiated and scored per repeat. Results are presented as a function of time post irradiation of either nucleus or cytoplasm, and unless specified, corrected for the control foci value in non-irradiated cells.

### Mitochondrial membrane polarisation measurement

Mitochondrial membrane polarisation can be assessed by measuring the retention of the tetramethylrhodamine ethyl ester perchlorate dye (TMRE, Sigma-Aldrich). When mitochondria are fully functional this cationic dye is preferentially taken up by the polarised, negatively charged mitochondria causing it to fluoresce red. However, when mitochondria undergo stress they lose their membrane potential becoming fully permeable meaning they no longer retain the dye and thereby lose fluorescence.

Considering that in the absence of GNP there is no significant mitochondria depolarisation^13^ the experiments were performed only on GNP treated cells. Tetramethylrhodamine ethyl ester perchlorate (TMRE) dye was added to each dish and incubated for 15 minutes prior to irradiation.

In different dishes, TMRE positive (polarised functional mitochondria) or TMRE negative (depolarised mitochondria) cells were irradiated with 2 Gy targeting the cytoplasm only and fixed 3 and 6 hours after irradiation. Control non-irradiated dishes were prepared and underwent the same treatment as the irradiated cells.

Flow cytometry was used to measure cell mitochondrial recovery after GNP removal. Samples were prepared as previously described^27^ using TMRE to detect depolarised mitochondria. TMRE fluorescence was analysed immediately using a FACSCalibur flow cytometer and CELL-Quest software (Becton-Dickson). 1 × 10^4^ cells were analysed per sample.

Negative controls (cells with depolarised mitochondria) were prepared and treated with 2.5 mM H_2_O_2_ for 30 minutes. Samples with no GNPs added were also prepared and treated as positive controls.

### GNP dose attenuation

To assess the physical impact of GNPs in this system, a simple cell was modelled using the Geant4 Monte Carlo toolkit as a hemispherical volume growing on a flat Mylar sheet, containing a spherical nucleus^28^. The dimensions of both the cell and the nucleus were selected using microscopy images of the cells under investigation. Irradiations with 278 eV photons were modelled, with doses calculated treating the nucleus and cytoplasm either as water, or water containing some concentration of gold. The attenuating effect of the GNPs was calculated as the ratio of nuclear integral dose between the with-gold and without-gold cases, to determine possible impact on physical dosimetry.

### Data analysis

DNA damage decay curves were fitted using the equation 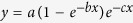 where a, b, c are free parameters, with average foci number calculated with subtraction of non-irradiated control values. AG01522 DNA damage kinetics following nucleus irradiation was fitted to a single exponential decay described by 
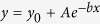
.

Flow cytometry data are presented as relative to untreated control values.

All data presented are means ± standard error of the mean. All experiments were performed for at least three independent repeats with at least 30 cells irradiated per data point for the microbeam irradiation. Flow cytometry experiments were done three times with 10 k events analysed per experiment. Data was fitted using Origin Pro 8.0 and statistical errors are calculated as the standard error of the mean. Statistical analysis for DNA damage experiments was carried out using Student’s t-test. Statistical significant differences were assumed at the level of *p* < 0.05.

## Results

### DNA Damage kinetics following subcellular irradiation

**Figure 2:**
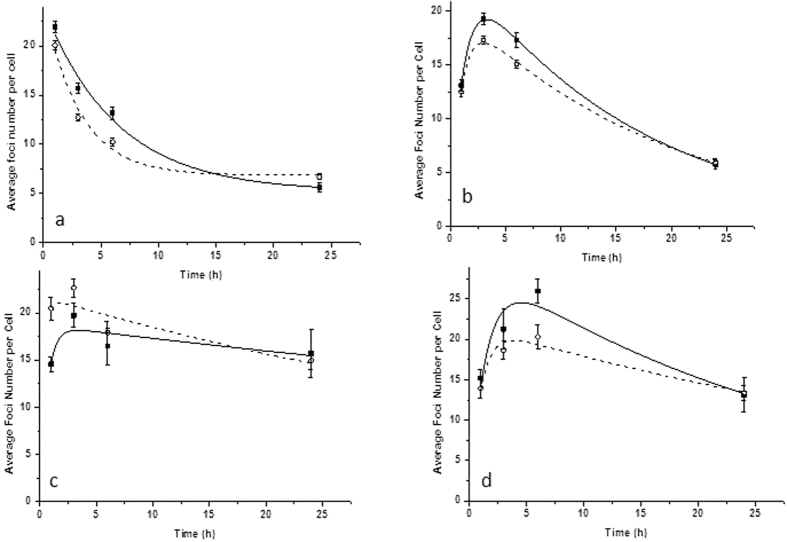
DNA damage kinetics (53BP1 foci assay) for AG01522 cell line (**a**) AG01522 nucleus irradiation, (**b**) AG01522 cytoplasm irradiation; and MDA-MB-231 cell line (**c**) MDA-MB231 nuclear, (**d**) MDA-MB-231 cytoplasm targeted irradiation with 2 Gy. Solid line represents fit for GNP treated cells (closed symbols), and dashed line represents control fit (open symbols). Average foci number is presented corrected for the control non-irradiated values; means are presented ± standard error of the mean n = 4.

In order to assess the impact of GNP on the DNA damage formation and repair, the 53BP 1 assay was used. Figure 2 shows 24 hr DNA damage repair kinetics for both cell lines after nucleus (Fig. 2a,c) and cytoplasm (Fig. 2b,d) targeted irradiation. Each plot highlights the impact of the presence of GNPs on the repair kinetic; data are corrected for non-irradiated controls. AG01522 cells show rapid DNA damage onset after nuclear irradiation (Fig. 2a) whilst foci induced by cytoplasmic irradiation reach a maximum three hours after irradiation as previously observed^29^. For the nucleus irradiated cells, the maximum average number of foci number is 21.9 ± 0.6 foci per cell for GNP treated cells and 20.1 ± 0.5 for untreated control (Fig. 2a).

In MDA-MB-231, nucleus irradiation yields maximum foci number after 3 hours in untreated cells with 19.7 ± 1.3 average foci per cell (Fig. 2c). In GNP treated cells, the levels of DNA damage reach a maximum of 22.66 ± 0.95 foci per cell after 3 hour incubation post irradiation.

After targeting the cytoplasm of the AG01522 cells, the DNA damage appears to reach a maximum level after 3 hours post irradiation, with a foci number of 17.3 ± 0.4 and 19.3 ± 0.5 without and with GNP respectively, as shown in Fig. 2b. The damage induced by cytoplasm irradiation peaks at 6 hours in MDA-MB-231 both the GNP treated cells and untreated cells, with 26.0 ± 1.5 and 20.3 ± 1.5 average foci per cell respectively (Fig. 2d). The presence of GNP causes significant (*p* < 0.05) differences in the maximum average foci number per cell in all cases, except for MDA-MB-231 nuclear irradiation.

**Figure 3:**
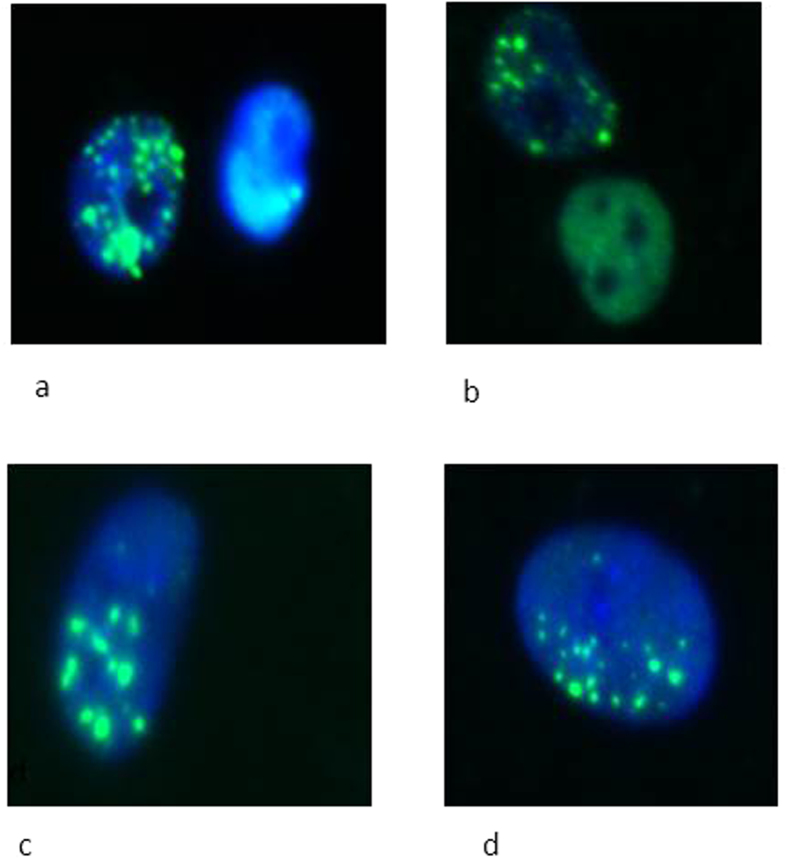
DNA damage shown as 53BP1 foci after microbeam targeted irradiation of the nucleus with 2 Gy and 1 hour post irradiation of (**a**) MDA-MB-231 cells treated with GNPs, (**b**) MDA-MB-231 cells not treated with GNPs; (**c**) AG01522 treated with GNPs and (**d**) non GNP treated AG01522 cells.

**Figure 4:**
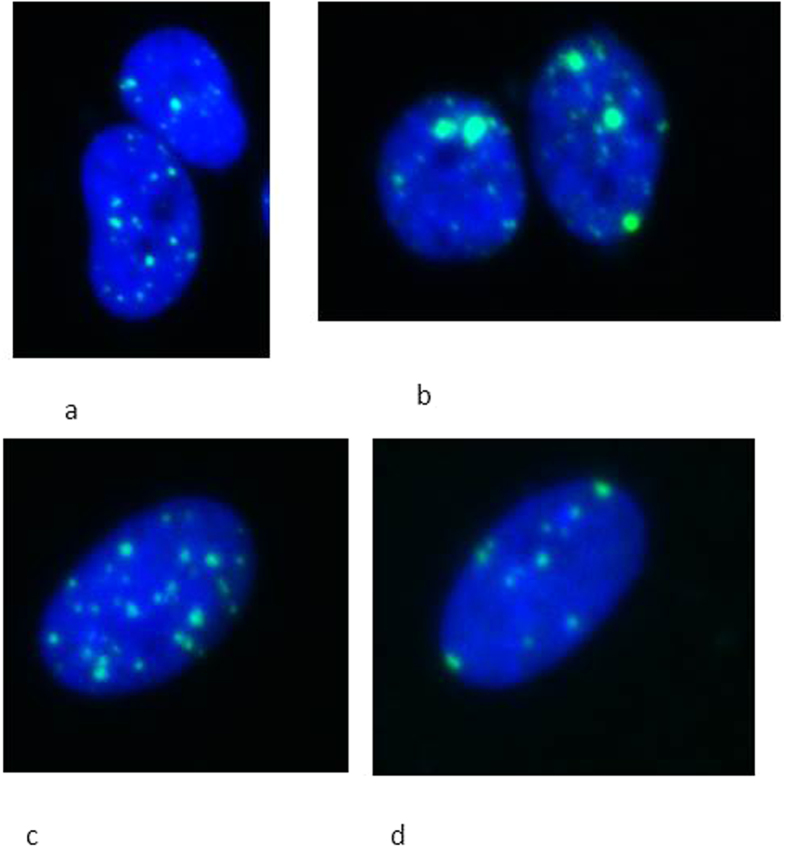
DNA damage shown as 53BP1 foci after microbeam targeted irradiation of the cytoplasm with 2 Gy and 3 hours post irradiation of (**a**) MDA-MB-231 cells treated with GNPs, (**b**) MDA-MB-231 cells not treated with GNPs; (**c**) AG01522 treated with GNPs and (**d**) non GNP treated AG01522 cells.

Figure 3 and Fig. 4 show the foci comparison after 1 and 3 hours post irradiation by 2 Gy of the nucleus and cytoplasm respectively for the investigated cell lines.

In both cell lines, DNA damage takes longer to accumulate after cytoplasmic irradiation, whether in the presence of nanoparticles or without. Once the DNA damage peaks, the breast adenocarcinoma cell line shows slow repair with considerable residual damage still present 24 hours after irradiation with a significantly more rapid disappearance seen in the normal fibroblast. The fitting parameters for Fig. 2 are presented in Table 1.

**Figure 5:**
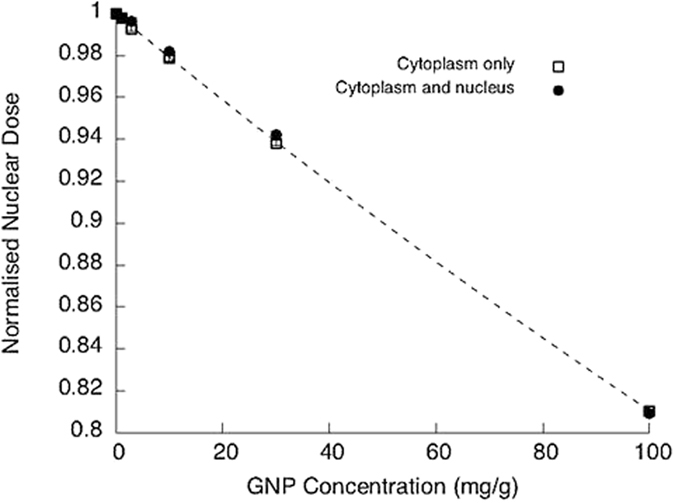
Nuclear dose calculation as a function of GNP concentration in the cytoplasm surrounding the nucleus. Simulations were performed for a 280 eV microbeam, 6 μm beam diameter irradiating cell nucleus. Two conditions were calculated: either for a given gold concentration throughout the cytoplasm and nucleus, or the same total amount of GNP constrained to the cytoplasm. Concentrations varied between 0.1 to 100 mg/g GNP.

Figure 5 shows the relative nuclear dose calculated as a function of the GNP concentration, assuming GNPs either in cytoplasm only or in cytoplasm and nucleus. This shows the photon absorption due to GNP presence in the cytoplasm assuming the beam travels across a thin layer of cytoplasm before it reaches the nucleus. At the energy used by the microbeam, 278 eV, gold’s attenuation coefficient is 55 times greater than water’s, therefore an individual 1.9 nm GNP causes a 5% attenuation of incident photons, compared to 0.1% in a similar volume of water. This means that, in contrast to higher energy X-ray irradiation, GNPs have an almost purely attenuating effect on these low-energy X-rays, and no physical dose enhancement is expected.

However, as seen below, GNP concentrations measured within these cells correspond to approximately 1% gold by mass, or 10 mg/g, which suggests the total absorption by the GNPs in the cytoplasm is only 2%, significantly less than the biological effects observed. This suggests that the effects observed are driven by chemical and/or biological mechanisms.

Previous work^14^ indicated AG01522 cell line as having the highest uptake of GNP of a panel of cell lines investigated. However, this did not translate into enhanced radiobiological effect, suggesting that biological processes have stronger influence in the overall effect than the physical dose enhancement.

**Figure 6:**
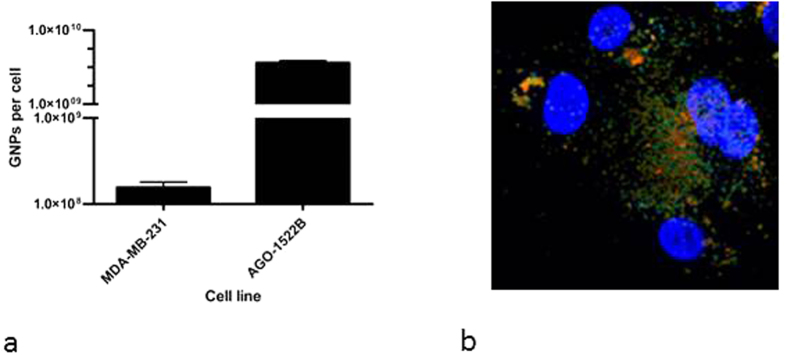
(**a**) 1.9 nm Aurovist™ nanoparticles cell uptake. The quantity of GNPs per cell was assessed after 24 hours incubation with 500 μg/mL GNPs by Intensity Coupled Plasmon Mass Spectrometry (ICP-MS). Means are presented ± standard error of the mean. N = 3. (**b**) FLIM and fluorescent microscopy of GNP uptake. MDA-MB-231 cells were treated with 1.9 nm GNPs for 24 hours. The slides were fixed and stained with an early endosomal antibody (green), with DAPI (nuclei) and GNPs (orange).

Figure 6a shows the uptake for the MDA-MB-231 cell line to be ~10^8^ GNPs per cell, whereas for the AG01522 cell line there would be ~10^9^ GNP per cell. These represent similar mass uptakes to other work in this field. For example, Chithrani *et al*.^12^ reported uptake of 2,000 GNPs of 100 nm diameter in HeLa cells, a mass equivalent to 3 × 10^8^ 1.9 nm GNPs. The GNP distribution throughout the cell was obtained by FLIMS, which shows the vast majority of the particles were found in the cytoplasm surrounding the nucleus, as seen in Fig. 6b. Once again, this is further evidence against the need for nuclear localisation of GNPs to induce DNA damage.

**Figure 7:**
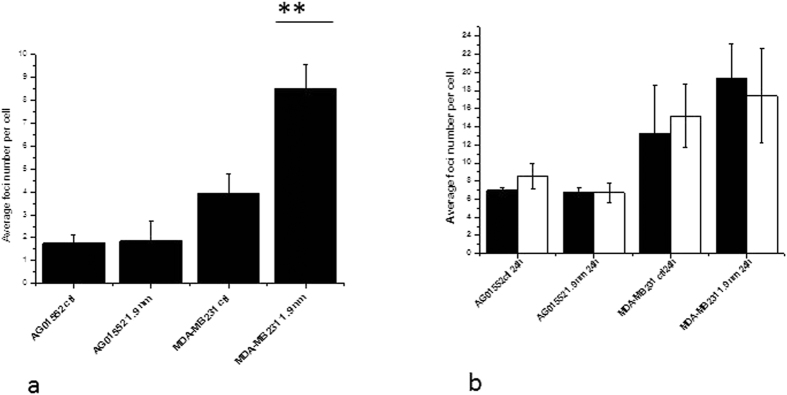
DNA damage as measured by fluorescence microscopy for 53BP1. (**a**) Non irradiated AG01522 and MDA-MB231 cells were compared with and without treatment with 1.9 nm Aurovist™ nanoparticles at 500 μg/mL for 24 hours; (**b**) 24 h residual foci levels after nucleus targeted irradiation (open columns) and cytoplasm irradiation (black columns) with a dose of 2 Gy. Average foci number is presented for four different repeats means are presented ± standard error of the mean (**p < 0. 01).

The cells lines experienced different toxicity levels as a consequence of GNP treatment alone, as shown in Fig. 7a, where MDA-MB-231 cells exhibit a significant increase in DNA damage following treatment with GNPs alone, while AG01522 show no significant effect. Similar effects were seen in residual damage, 24 hours after both nuclear and cytoplasmic irradiation as shown in Fig. 7b.

**Figure 8:**
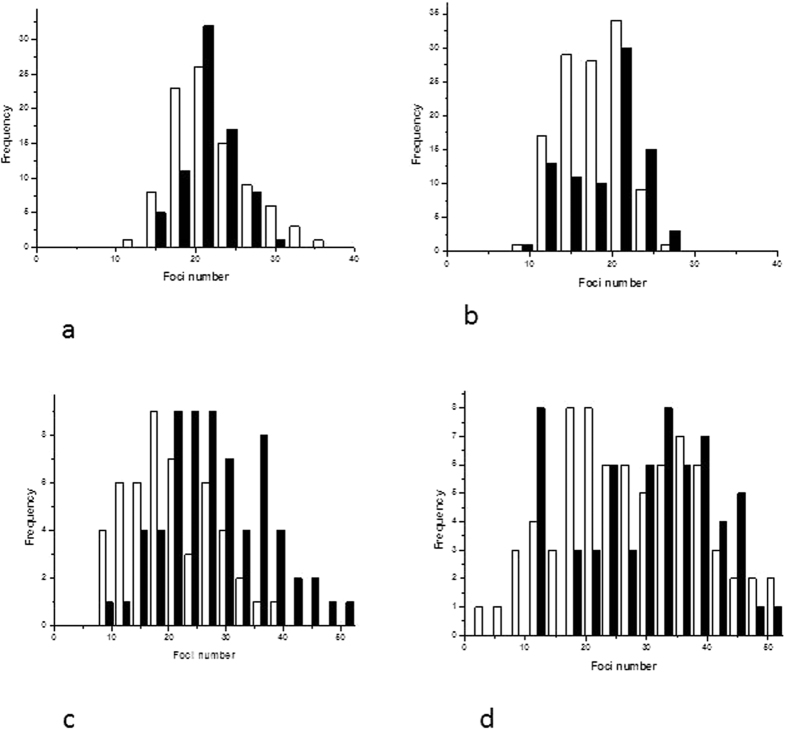
Distribution of foci per cells after targeted cytoplasm irradiation for AG01522 (**a**) 3 h and (**b**) 6 h and MDA-MD-231 (**c**) 3 h and (**d**) 6 h. Histogram show non-treated irradiated control as open columns and GNP treated 24 hours prior to cytoplasm targeted irradiation (black columns). This figure presents raw data not corrected for non-irradiated control foci values.

Figure 8 shows that increase in DNA damage following cytoplasm only irradiation appears to be a shift of the whole cell population. In contrast, the increase seen after 1.9 nm GNP and irradiation together appears to be a result of a sub-population of cells with an amplified level of DNA damage. This is particularly obvious in MDA-MB-231 cells at both 3 and 6 hours after irradiation and it is linked with previous mitochondrial polarisation work^13^. Figure 8 presents the raw data for all individual repeats and are not corrected for non-irradiated controls.

### Mitochondria polarisation studies

**Figure 9:**
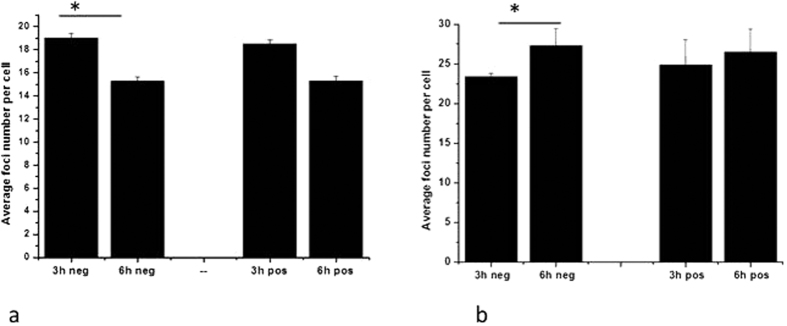
DNA damage following cytoplasm irradiation and GNP treated only cells after 3 and 6 hours incubation for (**a**) AG01522 and (**b**) MDA-MB-236. Cells were TMRE stained and irradiated as two different populations: TMRE negative with depolarised mitochondria and TMRE positive with functional mitochondria. Data is presented as means ± standard error corrected for the non-irradiated control foci numbers. n = 3 (*p < 0.05).

By combining TMRE fluorescent imaging with the microbeam platform we were able to target TMRE positive and TMRE negative cells within the same population of cells. As shown in Fig. 9, the foci numbers in AG01522 cells decrease between the two time points in agreement with the kinetics shown in Fig. 1. However in MDA-MB-231, TMRE negative cells show a significant increase in foci numbers between 3 and 6 hours post irradiation. The different behaviour between the cell lines points towards DNA damage detection stalling connected with the mitochondrial membrane status in the MDA-MB-231^29^.

**Figure 10:**
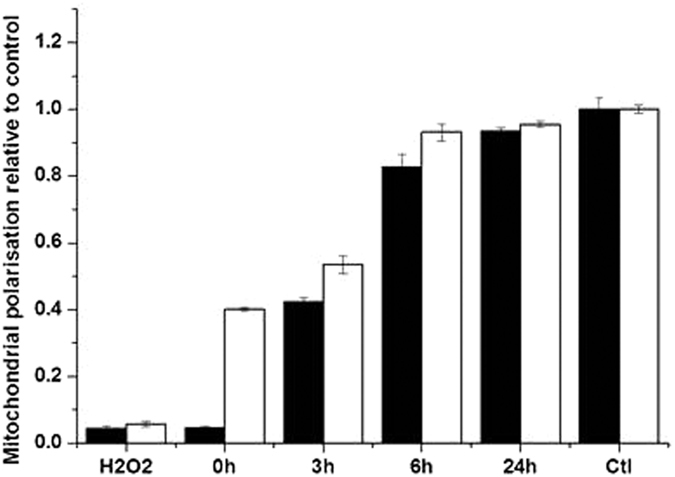
Mitochondrial polarisation after 24 h GNP incubation for AG01522 (open columns) and MDA-MB-231 (black columns) as measured by TMRE fluorescent flow cytometry. Time points represent incubation time after gold nanoparticles have been removed from the media. Cells were treated with 2.5 mM H_2_O_2_ for 30 minutes as a negative control. Means are presented ± standard error of the mean. n = 3.

Given the results of this study so far, mitochondrial polarisation recovery after the removal of GNP treatment became increasingly important in elucidating the DNA repair kinetics. Mitochondrial membrane polarisation was quantified at 0, 3, 6 and 24 hour time points after the removal of GNPs (Fig. 10) by measuring the TMRE fluorescence levels relative to control non-treated value. The gain of TMRE florescence across the cell lines after GNP removal indicates recovery of mitochondrial polarisation to the level of fluorescence corresponding to non GNP treated cells. As expected, MDA-MB-231 cells show a more dramatic mitochondrial depolarization down to less than 10% of control level when compared to the AG01522 at 0 h time point, which had a fluorescence level of 40%. The repolarisation kinetics also indicate the AG01522 cell line as fully recovered at the 6 h time point, whereas MDA-MB-231 cells show a similar recovery only after 24 h.

Mitochondrial membrane depolarisation, as shown in Fig. 10, is reversible upon removal of GNP treated media and replacement with normal media. However, as shown in previous work the temporary stalling of the mitochondrial function has long term effects at the cell survival level if radiation is delivered at this time^13^. This is in agreement with previous experiments showing no DNA damage detection after cytoplasm irradiation when mitochondrial function is impaired^29^.

## Discussion

The efficacy of radiotherapy is attributed largely to the damage it causes to nuclear DNA. In this study, for the first time we report the radiobiological response of cells following irradiation by nuclear or cytoplasmic targeting in the presence of GNPs.

Aurovist™ 1.9 nm gold nanoparticles have been extensively studied in the literature, being used in the first *in vivo* demonstrations of GNP radiosensitisation, and as such understanding their mechanism of action can provide insight into a range of experimental results.

Previously reported data show, after broad field 225 kVp irradiation, an increased level of foci in nanoparticle treated cells. However, the increased level in DNA damage present in specific cell lines is not related to radiosensitisation but it is more an extension of the GNP induced toxicity present before radiation treatment^13^. This observation in the context of the survival curves in the presence of GNP and uptake measurements previously published^30^ led to the investigations of effects not primarily driven by direct radiation damage to the DNA.

The challenge when working with radiosensitizing agents such as GNPs is in isolating the biological, chemical and physical effect. Previous simulation work^15^,^31^ investigating the effect of GNPs from the physical interaction point of view has established the physical dose enhancement as a function of incident energy, selecting an ideal energy interval for maximum biological effect in the kV domain. However, experimental studies^32^ at clinically relevant MV energies showed radiosensitization when compared with the non GNP treated controls irradiated at the same energy.

Consequently, it has been suggested that the physical effects of GNPs rely largely on Auger electrons being emitted after irradiation, with a range of a few hundred nanometers and which cause a series of clustered ionising events inducing a non-uniform dose deposition pattern around the nanoparticle^33^. These physical effects require GNPs to be delivered to the nucleus, or in close proximity. However, this work made use of an energy below that where significant physical effects are predicted for GNPs and where secondary electrons have ranges of less than ten nanometres^64^. Despite this, differences in response were observed when GNPs were added to cells, suggesting a biological or chemical mechanism of action.

Interestingly, these biological effects were most apparent not when the nucleus was directly irradiated, but rather when the cytoplasm was targeted, further highlighting the departure from the paradigm that nuclear targeting is most important in cellular response. Previous studies have suggested mitochondria as a key cytoplasmic mediator for both radiation damage and GNP sensitisation^29^. Mitochondria are often compromised under oxidative stress and, as key players in cell death the depolarisation of mitochondria can be detrimental to the DNA repair process and cell survival.

Even in the absence of GNPs, cells subjected to cytoplasmic irradiation show significantly more damage to the nucleus when the mitochondrial function is switched off^29^,^34^ suggesting a complex interplay between mitochondria, radiation damage, and the nucleus. In previously published clonogenic survival data^13^, GNP treatment combined with radiation increases the amount of cell kill compared with radiation alone even without nuclear localisation. These data also demonstrated that the introduction of GNPs impacts significantly on mitochondrial membrane polarisation and function, indicating a potential mechanism of radiation sensitisation^16^.

By specifically targeting the cytoplasm in these cells with low energy X-rays, the possibility of direct nuclear damage can be limited, allowing a more direct focus on the impact of cytoplasmic targets. This work clearly shows that disruption of the mitochondria using these 1.9 nm GNPs leads to increased nuclear damage as a result of cytoplasmic irradiation. Furthermore, TMRE fluorescence experiments provided details of how radiation affects the two distinct cells populations: one with fully functional and another with depolarised mitochondria. This showed in the MDA-MB-231 cells a delay in foci formation in the depolarised population, highlighting prolonged foci formation or a delay in the repair process thus causing a lower survival fraction after GNP treatment and irradiation.

These observations are highly relevant to the development of future nanoparticles. While there is a great interest in delivering GNPs to the nucleus through different coatings^23^, this work shows that even if the Aurovist™ nanoparticles do not permeate the nuclear membrane, they trigger biological effects with serious long term consequences. Even though the mitochondria depolarisation induced by the nanoparticles is a reversible effect, irradiating cells in this state gives cell survival significantly lower than the non-GNP treated cells.

When analysing the GNP uptake in both cell lines used in this work, AG01522 cells showed a significantly higher concentration of nanoparticles compared to MDA-MB-231 cells. However, foci levels for non-irradiated cells MDA-MB-231 showed significantly more toxicity, which was also reflected in the residual foci numbers at 24 hour post irradiation. This highlights that a higher GNP uptake will not necessarily translate into a greater dose enhancement, as seen in other studies^14^. This differential effect is an interesting characteristic of the Aurovist ™ GNPs, but whether this is applicable to other nanoparticles still needs to be determined.

The importance and complexity of these biological effects has significant impacts on attempts to optimise GNP design. GNPs come in a vast array of sizes and formulations, potentially capped with one of a large number of coatings and functionalisation. However, the impact different types of coating have on the cell cannot be easily estimated. Previous work^16^ has linked the radiosensitising properties of the Aurovist nanoparticles to its particular thiol coating due to evidence of its involvement in mitochondrial effects. This suggests that other coatings may drive effects through other processes, opening the opportunity to target a broad range of potential radiosensitising targets within the cell to deliver clinical benefit. The work presented here represents only a departure point for further investigations of GNPs. Based on this work, future studies can interrogate different outcomes such as the mitochondrial oxidation as a result of GNP action. Modifications of these particles, such as through the use of different coatings, surface charges or sizes may modulate these effects or potentially even drive entirely different processes, meaning simple extrapolations to novel particle types must be made with care.

## Conclusion

Using an X-ray microbeam to target sub-cellular regions with low energy X-rays, the radiosensitising effect of nanoparticles in the absence of any physical effects was quantified. This study highlighted that even in the absence of nuclear localisation, cytoplasmic damage can drive significant DNA damage, with mitochondria identified as a possible driver for gold nanoparticle radiosensitization, with an effect that is apparently cell-line specific. This study highlights new aspects of the mode of action of metal based nanoparticles and their utility as probes of radiation response at the cellular level.

## Additional Information

**How to cite this article:** Ghita, M. *et al*. A mechanistic study of gold nanoparticle radiosensitisation using targeted microbeam irradiation. *Sci. Rep.* **7**, 44752; doi: 10.1038/srep44752 (2017).

**Publisher's note:** Springer Nature remains neutral with regard to jurisdictional claims in published maps and institutional affiliations.

## Figures and Tables

**Table 1:** Tabulated values of fitting parameters for the DNA damage kinetics curves in Fig. 1.

Target	Parameter	Untreated control	GNP treated
AG01522 nucleus	y_0_	6.85 ± 0.94	5.21 ± 1.21
	A	17.47 ± 3.12	18.73 ± 2.45
	b	0.31 ± 0.08	0.15 ± 0.06
AG01522 cytoplasm	A	21.04 ± 0.4	25.40 ± 0.52
	b	0.99 ± 0.04	0.79 ± 0.03
	c	0.05 ± 0.001	0.06 ± 0.001
MDA-MB-231 nucleus	A	21.82 ± 2.42	18.76 ± 2.34
	b	3.09 ± 3.42	1.57 ± 0.73
	c	0.01 ± 0.005	0.008 ± 0.008
MDA-MB-231 cytoplasm	A	30.09 ± 4.7	21.88 ± 2.90
	b	0.67 ± 0.22	1.02 ± 0.36
	c	0.03 ± 0.01	0.02 ± 0.009
